# Physicians' awareness of non-pharmacological therapies for insomnia in the Czech Republic

**DOI:** 10.3325/cmj.2026.67.103

**Published:** 2026-04

**Authors:** Laura Hrehova Hantak, Norbert Král, Bohumil Seifert, Peter Kalanin

**Affiliations:** 1 Institute of General Practice, First Faculty of Medicine, Charles University, Prague, the Czech Republic; 2 Department of General Medicine, Faculty of Medicine, Pavol Jozef Šafárik University, Kosice, Slovakia

## Abstract

**Aim:**

To assess general practitioners’ (GP) awareness, attitudes, and practices related to non-pharmacological treatments for insomnia.

**Methods:**

A cross-sectional online survey was conducted between January and June 2024 among 100 general practitioners across urban and rural regions of the Czech Republic. The questionnaire assessed GPs’ awareness, attitudes, and practices related to non-pharmacological treatments for insomnia.

**Results:**

Most GPs (85%) reported using non-pharmacological approaches as the initial treatment, primarily sleep hygiene education (64%). Only 28% were familiar with Cognitive Behavioral Therapy for Insomnia, and fewer used structured diagnostic tools such as sleep diaries or validated scales. Hypnotics overuse was reported by 81% of respondents. Urban GPs were more likely to refer patients to sleep specialists and showed greater confidence in non-drug management. Patient interest in non-pharmacological therapies was moderate (31%), and adherence was influenced by the quality of the doctor-patient relationship.

**Conclusion:**

While Czech GPs are aware of non-pharmacological treatment options for insomnia, actual implementation – especially of CBT-I and diagnostic tools – is limited. Targeted training and better access to behavioral treatment resources could improve insomnia management in primary care and reduce reliance on pharmacological interventions.

Insomnia is a prevalent and complex sleep disorder that considerably affects quality of life, affecting an estimated 10% of adults. It is more common among primary care patients, with prevalence estimates ranging from 10% to 20% (1,2). Symptoms include difficulty falling asleep, staying asleep, or waking up too early, along with daytime fatigue, mood disturbances, and cognitive difficulties. In many cases, insomnia can also occur alongside other medical or psychiatric disorders, which complicates diagnosis and treatment. Untreated insomnia can contribute to the development or exacerbation of different conditions, which makes it a critical issue for primary care providers. General practitioners (GPs) are often the first point of contact for patients experiencing insomnia (3). They play a key role in assessing the nature of the disorder, using tools such as the Insomnia Severity Index or the Pittsburgh Sleep Quality Index to evaluate its severity (4,5). The prevalence of insomnia in primary care patients was reported to be 69%, with 50% reporting occasional insomnia and 19% reporting chronic insomnia (6). However, there is limited awareness and implementation of evidence-based non-pharmacological treatments such as Cognitive Behavioral Therapy for Insomnia (CBT-I) in general practice (7). CBT-I is widely recognized as the most effective treatment for insomnia, with the American Academy of Sleep Medicine and the European Sleep Research Society guidelines recommending its use as the first-line approach, especially for chronic insomnia (8,9). In the Czech Republic, as well as in other countries, GPs face various challenges in incorporating non-pharmacological treatments into their practice, including lack of training, resource limitations, and time constraints. While pharmacological interventions, including hypnotic medications, are often prescribed, research supports a broader use of CBT-I and other behavioral approaches (10). Identifying gaps in physician knowledge, training, and referral systems is crucial for improving the management of insomnia in primary care settings. Additionally, integrated care models involving collaboration between GPs, sleep specialists, and behavioral therapists may improve treatment adherence and long-term management of insomnia. A collaborative care model for insomnia led to improved treatment adherence and more effective long-term management compared with traditional care (11). Despite growing international evidence supporting non-pharmacological treatments for insomnia, there is a lack of data on how these approaches are understood and implemented by general practitioners in the Czech Republic. Previous research has primarily focused on treatment efficacy or patient outcomes, while limited attention has been given to physicians’ awareness, attitudes, and real-world clinical practices in primary care settings. This study addresses this gap by providing country-specific data on Czech general practitioners, with a particular focus on the discrepancy between guideline recommendations and actual clinical practice.

The aim of this study is to assess the awareness of non-pharmacological treatments for insomnia among GPs in the Czech Republic. Specific aims included examining the knowledge of GPs regarding evidence-based non-pharmacological therapies for insomnia, assessing the frequency and barriers to implementation of non-pharmacological treatments in routine practice, exploring GPs' prescribing practices, particularly concerning the use of pharmacological treatments, and investigating the impact of patient demographics on the management of insomnia.

## Participants and methods

### Design

The research was conducted from January to June 2024. The study protocol was approved by the Ethics Committee of the General Teaching Hospital in Prague. The surveys were distributed online to GPs listed in the contact database provided by the Society of General Practitioners in the Czech Republic, which ensured a targeted and comprehensive outreach to eligible participants across the country.

The questionnaire (Supplemental Material(Supplementary Material)) was specifically developed by the authors for the purposes of this research, based on a review of relevant literature and existing clinical guidelines related to the non-pharmacological treatment of insomnia. As the questionnaire was newly designed, it was not previously validated. However, to enhance content validity, the initial version was reviewed by three experts in general practice and sleep medicine, and subsequently piloted on a small sample of general practitioners (n = 10) to assess clarity and comprehensibility. Feedback from the pilot testing was used to revise the final version.

All participants were required to comfortably understand the Czech language and have access to the internet. The first page of the web questionnaire contained the consent form, and participants had the right to withdraw from the survey at any time. The survey platform ensured secure data collection and protection.

### Participants

The study participants were selected from a pool of actively practicing GPs in both urban and rural regions of the Czech Republic. A stratified sampling was used to ensure a representative distribution of GPs from both urban and rural areas. The sample was designed to reflect the population distribution across different regions. Urban areas were defined as regions with larger population density, and rural areas as those with smaller population sizes and more geographically dispersed settlements. Participants were selected through postal code data, and we made sure that the sample included a proportionate number of GPs from urban and rural regions according to the overall demographic distribution of the population. To ensure a broad and diverse sample, no specific criteria were imposed regarding the size of the medical practices, allowing for a range of practice types from small rural clinics to large urban health centers. Exclusion criteria included being unemployed, retired, or on pregnancy/parental leave at the time of the survey. This helped ensure that the participants were representative of those who regularly engage with patients and are involved in the direct management of health conditions, including insomnia.

### Data collection

Data were collected through an anonymous web-based survey using the Survio survey tool. The survey was distributed to 500 GPs, yielding 100 completed responses (response rate of 20%). The questionnaire consisted of 24 questions, addressing sociodemographic variables (sex, age), education level, practice size, and professional performance. Additionally, specific questions addressed the management of insomnia therapy in primary care practice.

### Statistical analysis

Frequency tables and contingency tables were used, and for the description of numerical variables, basic numerical characteristics (average, median, mode, standard deviation, minimum and maximum) were used. The χ^2^ test was used to test the association between categorical variables. The Mann-Whitney test was used to assess the differences between independent samples for ordinal variables. The Spearman correlation test was used to assess correlations between two continuous or ordinal variables. The significance level was set at *P* = 0.05. Statistical analysis was conducted using the statistical package Jamovi.

## Results

A total of 100 responses were received. Most respondents were female (84%). The sociodemographic characteristics of the participants are presented in Table 1.

**Table 1:** Demographic characteristics of respondents

Variable	n
**Gender**	
female	84
male	16
**Age category (years)**	
<35	35
36-45	45
46-55	12
56-65	6
>65	2
**Specialization**	
without attestation	25
certified general practitioner	75
**Type of practice**	
solo practice	54
group practice (without shared records)	11
group practice (with shared records)	35
**Practice location size**	
>100 000 inhabitants	43
50 000–100 000	18
<50 000	23
rural area	16
**Region of practice**	
Prague	35
Central Bohemia	10
Hradec Králové	8
South Moravian	8
South Bohemian	6
Ústí nad Labem	6
Moravian-Silesian	5
Olomouc	5
Zlín	5
Pardubice	4
Vysočina	3
Liberec	3
Plzeň	2
**Number of registered patients**	
≤1000	4
1001-1500	17
1501-2000	42
>2001	37

### Frequency of GP visits for sleep disorders

Patients with sleep disorders were seen by 36% of GPs 0-1 times per week, by 48% 2-3 times per week, and by 16% more than 3 times per week. Younger GPs (≤35 years) significantly more frequently reported low consultation frequency (0-1 × /week), whereas older GPs (>35 years) more often reported higher consultation frequency (2-3 × and >3 × /week) (*P* = 0.005). No significant association was found for attestation status (*P* = 0.065) or city size (*P* = 0.060) (Table 2). The number of registered patients did not influence how frequently a GP encountered a patient with a sleep disorder in their practice.

**Table 2:** Frequency of consultations for sleep disorders in general practice by age, attestation status, and city size*

Variable	0-1 × /week (n = 36)	2-3 × /week (n = 48)	>3 × /week (n = 16)	Total (n = 100)	χ^2^	p
**Age (years)**					10.4701	0.005
>35	16 (44.44)	37 (77.08)	12 (75.00)	65 (65.00)		
≤35	20 (55.56)	11 (22.92)	4 (25.00)	35 (35.00)		
**Attestation**					5.4815	0.065
yes	23 (63.89)	41 (85.42)	11 (68.75)	75 (75.00)		
no	13 (36.11)	7 (14.58)	5 (31.25)	25 (25.00)		
**City size**					5.6268	0.060
≤50 000 inhabitants	16 (44.44)	21 (43.75)	2 (12.50)	39 (39.00)		
>50 000 inhabitants	20 (55.56)	27 (56.25)	14 (87.50)	61 (61.00)		

*Data are shown as n (%).

### Patient characteristics and therapy approaches

In total, 20.3% of GPs reported referring patients to specialist sleep clinics, with urban areas showing a higher referral rate than rural areas (*P* = 0.004). Referral rates did not differ significantly according to GP sex or attestation status. GPs reported that insomnia was more commonly observed in female patients (76%) than in male patients (6%), while 18% of cases were unspecified. The highest prevalence of insomnia was reported in patients aged >65 years, followed by those aged 45-65 years. Regarding treatment approach, 85% of GPs reported initiating therapy with non-pharmacological methods, while 15% initiated it with pharmacological treatment. No significant age-related differences were observed in the initial treatment approach (*P* = 0.106) (Table 3).

**Table 3:** Patients‘ gender and therapy approaches by general practitioner‘s age

Variable	≥35 y (n = 65)	<35 y (n = 35)	Total (n = 100)	*P* value
**Gender**				0.059
male	2 (3.08)	4 (11.43)	6 (6.0)	
female	54 (83.08)	22 (62.86)	76 (76.0)	
not specified	9 (13.85)	9 (25.71)	18 (18.0)	
**Initial treatment approach**				0.106
non-pharmacological	58 (89.23)	27 (77.14)	85 (85.0)	
pharmacological	7 (10.77)	8 (22.86)	15 (15.0)	

### Hypnotics use in insomnia treatment

A total of 81% of GPs reported encountering patients who overused hypnotics or sedatives, which was considered an important issue in their practice. No significant differences were observed based on sex (*P* = 0.504), attestation status (*P* = 0.105), city size (*P* = 0.176), or age group (*P* = 0.298) (Table 4).

**Table 4:** Hypnotics overuse in insomnia treatment by gender, attestation, city size, and age*

	Hypnotics overuse				
Variable	yes (n = 81)	no (n = 19)	Total (n = 100)	χ^2^	p
**Gender**				0.4456	0.504
male	12 (14.81)	4 (21.05)	16 (16.00)		
female	69 (85.19)	15 (78.95)	84 (84.00)		
**Attestation status**				2.6207	0.105
yes	58 (71.60)	17 (89.47)	75 (75.00)		
no	23 (28.40)	2 (10.53)	25 (25.00)		
**City size**				1.8322	0.176
≤50 000 inhabitants	29 (35.80)	10 (52.63)	39 (39.00)		
>50 000 inhabitants	52 (64.20)	9 (47.37)	61 (61.00)		
**Age (years)**				1.0818	0.298
≤64	28 (34.57)	9 (47.37)	37 (37.00)		
>64	53 (65.43)	10 (52.63)	63 (63.00)		

*Data are shown as n (%).

### Education about non-pharmacological treatment of insomnia

Overall, 59% of GPs reported that they were able to educate patients about non-pharmacological treatment of insomnia, while 40% reported providing such education to a limited extent and 1% not at all. No significant differences were observed based on age (*P* = 0.359) or attestation status (*P* = 0.565) (Table 5).

**Table 5:** Education about non-pharmacological treatment of insomnia by general practitioner’s (GP) age and attestation status*

	GP able to educate patients about non-pharmacological treatment of insomnia					
Variable	yes (n = 59)	limited yes (n = 40)	no (n = 1)	Total (n = 100)	χ^2^	p
**GP age (years)**					2.0507	0.359
≥35	41 (69.49)	23 (57.50)	1 (100.00)	65 (65.00)		
<35	18 (30.51)	17 (42.50)	0 (0.00)	35 (35.00)		
**Attestation status**					1.1435	0.565
yes	46 (77.97)	28 (70.00)	1 (100.00)	75 (75.00)		
no	13 (22.03)	12 (30.00)	0 (0.00)	25 (25.00)		

*Data are shown as n (%). Percentages are calculated within columns.

### Use of diagnostic tools and approaches to insomnia treatment

The majority of GPs did not use formal sleep-related questionnaires in clinical practice. Only a small percentage used tools like sleep diaries, the Sleep Hygiene Index, or the Epworth Sleepiness Scale (Table 6). The majority of GPs were familiar with non-pharmacological treatments for insomnia, particularly Sleep Hygiene Guidelines (64%), exercise (44%), and phytotherapy (43%). Other approaches, such as cognitive behavioral therapy, phototherapy, and music therapy, were less commonly recognized, while even fewer GPs were familiar with massage/acupuncture/acupressure or autogenic training (Table 7).

**Table 6:** Use of sleep-related questionnaires in clinical practice

Questionnaire	n
None	81
Sleep diary	8
Sleep Hygiene Index	7
Epworth Sleepiness Scale	4
Pittsburgh Sleep Quality Index	3

**Table 7:** General practitioners’ awareness of non-pharmacological treatment methods*

Method	n
Sleep hygiene	64
Exercise	44
Phytotherapy	43
Aromatherapy	34
Cognitive behavioral therapy	28
Phototherapy	24
Music therapy	24
Massage/acupuncture/acupressure	13
Autogenic training	10

*Multiple responses were allowed.

### Patient feedback on non-pharmacological treatments

Patients reported valuing the non-pharmacological approaches recommended by their GPs, with Sleep Hygiene Guidelines being the most appreciated (67%), followed by cognitive behavioral therapy (20%) and exercise (11%). When providing feedback on treatment, 23% of patients viewed non-pharmacological treatments positively, 30% disapproved of them and preferred medication, and another 30% were neutral.

### Familiarity with sleep hygiene rules and effectiveness of non-pharmacological vs pharmacological approaches

The majority of GPs were familiar with sleep hygiene principles, including environmental modifications, avoidance of stimulants, and maintaining a consistent sleep schedule (Table 8). Among non-pharmacological approaches, sleep hygiene was the most commonly recommended strategy. Regarding pharmacological treatment, 19% of GPs considered it effective, 59% somewhat effective, and 14% somewhat ineffective. Approximately 31% of patients expressed interest in non-pharmacological treatment options.

**Table 8:** General practitioners’ familiarity with sleep hygiene rules

Sleep hygiene rule	n
Minimize noise and light; maintain a cool room temperature (18-20°C)	94
Avoid caffeine (coffee, tea, cola, energy drinks) in the late afternoon/evening	93
Avoid alcohol in the evening	91
Use the bed only for sleep and sexual activity	89
Avoid heavy meals in the evening (last meal 3-4 h before bedtime)	87
Maintain a consistent sleep schedule (±15 min)	85
Limit time spent in bed; avoid lying awake unnecessarily	80
Light physical activity (eg, walking) is beneficial; avoid exercise before bedtime	77
Avoid stressful discussions in the evening	77
Avoid smoking before sleep and during night awakenings	71

*Multiple responses were allowed.

## Discussion

In our study, insomnia was reported to be more common in female and older patients, and GPs in urban areas were more likely to refer patients to specialist care, while most patients were managed within primary care. The majority of GPs reported initiating treatment with non-pharmacological approaches, particularly sleep hygiene; however, the use of structured diagnostic tools and cognitive behavioral therapy remained limited. At the same time, pharmacological treatments remain part of clinical practice, and a high proportion of GPs reported encountering overuse of hypnotic medications. Younger GPs tended to engage less frequently with insomnia patients. Other studies also suggest a gap in knowledge or confidence in managing sleep disorders among younger doctors. For instance, younger practitioners felt less equipped to manage chronic conditions such as insomnia due to limited exposure in their early training (12,13).

Interestingly, we found no significant difference in referral rates based on GP’s attestation status. Similarly, Rubinstein et al (14) found that the certification status of a GP had little bearing on the treatment outcomes for sleep disorders.

In our study, urban areas showed higher referral rates to specialist sleep clinics. Previous studies also suggested that urban GPs had better access to sleep medicine resources and continuous professional development opportunities (14,15).

The majority of GPs in our study reported initiating treatment with non-pharmacological methods. This is in line with current clinical guidelines, which recommend cognitive-behavioral therapy for insomnia as the first-line treatment. Given that long-term hypnotics use is associated with development of dependence and tolerance, there is a global shift toward non-pharmacological approaches. However, this shift is not universally adopted. Despite guideline recommendations, many GPs hesitate to prescribe non-pharmacological treatments due to concerns over their efficacy and patient reluctance (16).

In a study by Skjeie et al, the majority of the 11 GPs interviewed reported having more than 10 older patients prescribed hypnotics for daily use, with a similar number receiving them on an intermittent basis (17). In fact, elderly patients are more likely to misuse sedatives due to polypharmacy or lack of education on alternative therapies (18).

Janus et al found that patients preferred medications to non-pharmacological treatments, emphasizing the need for better patient education on alternative therapies (19). According to Glass et al, older patients often sought hypnotics due to immediate symptom relief, even when they were aware of the potential side effects (20). Although GPs highly value non-pharmacological treatments, they face a significant challenge in patient adherence. In our study, only 31% of patients were interested in non-pharmacological rather than medication treatment. Patient preferences for medication often outweigh the benefits of non-pharmacological therapies, particularly in the context of a poor GP-patient relationship (21). Countries with stronger primary care systems – such as those in Scandinavia –benefit from higher continuity of care and more stable doctor-patient relationships, which are associated with improved adherence to treatment recommendations, including non-pharmacological interventions (22).

While a considerable proportion of GPs reported being able to educate patients about non-pharmacological therapies, actual patient engagement with these approaches remained inconsistent. Although GPs are aware of such treatments, they frequently encounter limited consultation time, patient skepticism, and insufficient training or resources to effectively implement non-pharmacological interventions (23-25). Although CBT-I is considered the gold standard for insomnia, its adoption in primary care is limited. Aschim et al reported that, despite recognized benefits of CBT-I, the therapy remained underutilized in primary care. Key barriers include limited time to master techniques, insufficient patient load suitable for CBT-I, lack of supervision during clinical hours, and inadequate financial incentives (26,27). Our study also revealed that although many GPs were familiar with non-pharmacological approaches such as sleep hygiene, CBT-I remained underutilized compared with other treatments. In contrast, nations with more integrated sleep-medicine training – such as the US, where primary-care providers receive structured CBT-I education, and the UK, with National Health Systems Improving Access to Psychological Therapies offering systematic referral and trained staff – demonstrate greater CBT-I adoption in clinical settings, compared with regions without such frameworks (24,28).

Our study also highlights a significant gap in the use of diagnostic tools: the majority of GPs did not employ standardized questionnaires like the Epworth Sleepiness Scale. Campbell et al found that ESS scores were frequently omitted in primary-care referrals for suspected sleep apnea (29). This lack of formal assessment methods may lead to underdiagnosis or misdiagnosis of insomnia, possibly contributing to overreliance on pharmacological treatments rather than on more evidence-based, non-pharmacological options. Israel and Lieberman emphasize that primary-care physicians often lack clear diagnostic criteria, face time constraints, and have limited training in sleep medicine – factors that promote reliance on sedatives (30).

In our study, 64% of GPs were familiar with sleep hygiene principles and often used them as a first-line recommendation. Sleep hygiene is widely regarded as an effective and easily implemented non-pharmacological intervention. Indeed, a UK survey found that 88% of GPs provided sleep hygiene advice for insomnia, despite often resorting to medication because they viewed it as insufficient on its own (13). However, consistent application of sleep hygiene principles remains challenging and depends heavily on the clinician’s training, confidence in managing insomnia, and available consultation time (31). Further, in our study, only 23% of patients viewed non-pharmacological treatments positively. Similarly, Haskard Zolnierek and DiMatteo demonstrated that better physician-patient communication was associated with significantly better treatment adherence, suggesting that poor rapport may reduce patient engagement with non-pharmacological sleep interventions (32).

In line with previous studies, our research also points to a greater awareness of potential benefits of non-pharmacological treatments in larger cities, where GPs are more likely to have access to training and resources for implementing these approaches. This situation also creates a disparity in care between urban and rural areas, as rural GPs often lack sufficient resources to support non-pharmacological treatments, contributing to the higher rates of hypnotic prescriptions observed in these regions (33,34).

This study has several limitations. The relatively small sample size and low response rate may reduce the generalizability of the findings and introduce potential selection bias. In addition, as the study was conducted within a single national health care setting, the applicability of the findings to other health care systems may be limited.

In conclusion, our findings indicate variability in the implementation of evidence-based non-pharmacological approaches in primary care. Further education and improved access to behavioral sleep interventions may support more consistent and effective management of insomnia. While Czech GPs are aware of non-pharmacological treatment options, their routine implementation in clinical practice remains inconsistent.
