# Supplementary material for: Physicians' awareness of non-pharmacological therapies for insomnia in the Czech Republic
Source: Croat Med J. 2026 Apr;67(2):103–11. doi: 10.3325/cmj.2026.67.103 (PMC13176932; doi:10.3325/cmj.2026.67.103)
Supplement: Supplementary Material [file CroatMedJ_67_s003.pdf]

Supplementary Material

Questionare

1 Gender
Select one answer

- male
- female

2 Age
Select one answer

- up to 35 years (inclusive)
- 36–45 years
- 46–55 years
- 56–65 years
- over 65 years

3 Specialization
Select one answer

- without certification
- certification in general practice (GP)

4 What type of practice do you work in?
Select one answer

- Solo practice (practice run by a single physician)
- Group practice (two or more physicians sharing premises but not sharing patient records)
- Group practice (two or more physicians sharing a common patient record system)
- Other option

5 I primarily practice in
Select one answer

- city with over 100,000 inhabitants
- city with 50,000–100,000 inhabitants
- city with up to 50,000 inhabitants
- rural area

6 Location
Select one answer

- Prague
- Central Bohemian Region (excluding Prague)
- Plzeň Region
- Karlovy Vary Region

- Hradec Králové Region
- Liberec Region
- Ústí nad Labem Region
- South Bohemian Region
- Pardubice Region
- Vysočina Region
- South Moravian Region
- Zlín Region
- Olomouc Region
- Moravian-Silesian Region

7 Number of registered patients in the practice
Select one answer

- up to 1,000 registered patients
- up to 1,500 registered patients
- up to 2,000 registered patients
- over 2,000 registered patients

8 I manage patients with sleep disorders (problems with falling asleep, staying asleep, or sleep quality)
Select one answer

- 0–1 times per week
- 2–3 times per week
- more than 3 times per week
- other

9 In my practice, I observe that insomnia is more common in
Select one answer

- men
- women
- I don’t know

10 I observe that insomnia is more common in the age group
Select one answer

- up to 30 years
- 31–44 years
- 45–64 years
- seniors over 65 years

11 If I refer a patient to a specialized workplace, it is mainly to
Select one or more answers

- Neurology
- Sleep medicine department/center
- Psychology
- Psychiatry
- I rather do not refer
- other…

12 What percentage of patients with insomnia do I refer to a specialized workplace
Select one or more answers

- 10%
- 20%
- 30%
- 40%
- 50%
- 60%
- 70%
- 80%
- 90%
- 100%

13 When managing a patient with insomnia, I usually start therapy with
Select one answer

- referral to a specialized workplace
- initiating pharmacotherapy
- non-pharmacological approaches
- other…

14 I am able to educate patients about non-pharmacological approaches to insomnia treatment
Select one answer

- no
- yes
- limited yes

15 I encounter that patients overuse hypnotics/sedatives in insomnia treatment
Select one answer

- no

- yes
- I don’t know

16 Do you consider the overuse of hypnotics/sedatives by patients a problem in your practice?
Select one answer

- yes
- no
- I don’t know
- other answer

17 When managing a patient with insomnia, I use one of the questionnaires
Select one or more answers

- Pittsburgh Sleep Quality Index
- Sleep Hygiene Index
- keeping a sleep diary
- Epworth Sleepiness Scale
- I usually do not use any
- I use others

18 From non-pharmacological approaches to insomnia treatment, I am familiar (at least passively) with
Select one or more answers

- Cognitive behavioral therapy
- Ten rules of sleep hygiene
- Phototherapy
- Music therapy
- Aromatherapy
- Exercise
- Massage/acupuncture/acupressure
- Phytotherapy
- Autogenic training
- I do not know any
- other…

19 From non-pharmacological approaches to insomnia treatment, patients report the greatest benefit (in terms of effect)
Select one or more answers

- Cognitive behavioral therapy
- Ten rules of sleep hygiene

- Phototherapy
- Music therapy
- Aromatherapy
- Exercise
- Massage/acupuncture/acupressure
- Phytotherapy
- Autogenic training
- other…

20 Based on feedback, patients evaluate non-pharmacological approaches as
Select one answer

- very positive
- rather positive
- neutral
- rather negative
- very negative

21 From the ten rules of sleep hygiene, I know the statement
Select one or more answers

- I do not know any
- From late afternoon (4–6 hours before bedtime), do not drink coffee, black or green tea, cola, or energy drinks, and limit their intake during the day. They are stimulating and disrupt sleep.
- Avoid heavy meals in the evening and have your last meal 3–4 hours before bedtime.
- A light walk after dinner may improve your sleep. On the contrary, exercising 3–4 hours before bedtime may disrupt sleep.
- After dinner, avoid dealing with important issues that may upset you. Instead, try to relieve stress with pleasant activities and prepare for sleep.
- Do not drink alcohol in the evening to fall asleep more easily – alcohol worsens sleep quality.
- Do not smoke, especially before falling asleep or during nighttime awakenings. Nicotine also stimulates the body.
- Use the bed and bedroom only for sleep and sexual activity (remove the TV from the bedroom; do not eat, read, or rest in bed during the day).
- Minimize noise and light in the bedroom and ensure a suitable temperature (ideally 18–20°C).
- Go to bed and wake up at the same time every day (including weekends) ± 15 minutes.
- Limit time spent in bed to the necessary minimum. Do not lie in bed unnecessarily; the bed is not for thinking.

22 Which non-pharmacological method in insomnia treatment has proven most effective for you?
Select one or more answers

- Cognitive behavioral therapy
- Ten rules of sleep hygiene
- Phototherapy
- Music therapy
- Aromatherapy
- Exercise
- Massage/acupuncture/acupressure
- Phytotherapy
- Autogenic training
- I don’t know
- none

23 Do you agree with the statement that non-pharmacological approaches to insomnia treatment are effective in practice?
Select one answer

- yes
- rather yes
- I don’t know
- rather no
- no

24 What percentage of patients suffering from insomnia are interested in addressing insomnia in a way other than medication (i.e., non-pharmacologically)
Select one or more answers

- 10%
- 20%
- 30%
- 40%
- 50%
- 60%
- 70%
- 80%
- 90%
- 100%
